# Modulation of hERG K^+^ Channel Deactivation by Voltage Sensor Relaxation

**DOI:** 10.3389/fphar.2020.00139

**Published:** 2020-02-28

**Authors:** Yu Patrick Shi, Samrat Thouta, Thomas W. Claydon

**Affiliations:** Department of Biomedical Physiology and Kinesiology, Simon Fraser University, Burnaby, BC, Canada

**Keywords:** hERG, relaxation, voltage sensor, gating, deactivation, mode-shift

## Abstract

The hERG (human-ether-à-go-go-related gene) channel underlies the rapid delayed rectifier current, I_kr_, in the heart, which is essential for normal cardiac electrical activity and rhythm. Slow deactivation is one of the hallmark features of the unusual gating characteristics of hERG channels, and plays a crucial role in providing a robust current that aids repolarization of the cardiac action potential. As such, there is significant interest in elucidating the underlying mechanistic determinants of slow hERG channel deactivation. Recent work has shown that the hERG channel S4 voltage sensor is stabilized following activation in a process termed relaxation. Voltage sensor relaxation results in energetic separation of the activation and deactivation pathways, producing a hysteresis, which modulates the kinetics of deactivation gating. Despite widespread observation of relaxation behaviour in other voltage-gated K^+^ channels, such as *Shaker*, Kv1.2 and Kv3.1, as well as the voltage-sensing phosphatase *Ci*-VSP, the relationship between stabilization of the activated voltage sensor by the open pore and voltage sensor relaxation in the control of deactivation has only recently begun to be explored. In this review, we discuss present knowledge and questions raised related to the voltage sensor relaxation mechanism in hERG channels and compare structure-function aspects of relaxation with those observed in related ion channels. We focus discussion, in particular, on the mechanism of coupling between voltage sensor relaxation and deactivation gating to highlight the insight that these studies provide into the control of hERG channel deactivation gating during their physiological functioning.

## Overview

The mechanisms by which membrane depolarization triggers the voltage sensing unit to undergo conformational changes that lead to opening of the voltage-gated potassium (Kv) channel pore are of significant interest. Recent findings have shown that prolonged activation of Kv channels leads to further reconfiguration of the S4 voltage sensor into a stable activated relaxed state. This relaxation imparts hysteresis, or mode-shift behavior, where the voltage-dependence of S4 return and subsequent pore closure occurs at more hyperpolarized membrane potentials than are required to activate S4 and open the pore. Voltage sensor relaxation was recently demonstrated in hERG channels, and is likely a contributor to slow deactivation gating. The significance of relaxation and stabilization of the activated hERG voltage sensor have only recently begun to be recognized as potential orchestrators of the unique and critical I_Kr_ repolarizing current during the cardiac action potential. This review aims to highlight this role by describing current understanding of the role of voltage sensor relaxation in the slow deactivation gating of hERG channels. To begin, we first provide a brief overview of the structure-function aspects of hERG channels, followed by detailed review of the mechanistic determinants of voltage sensor relaxation and its connection to slow deactivation gating in hERG channels.

### Structure of the hERG Channel

**Figure 1:**
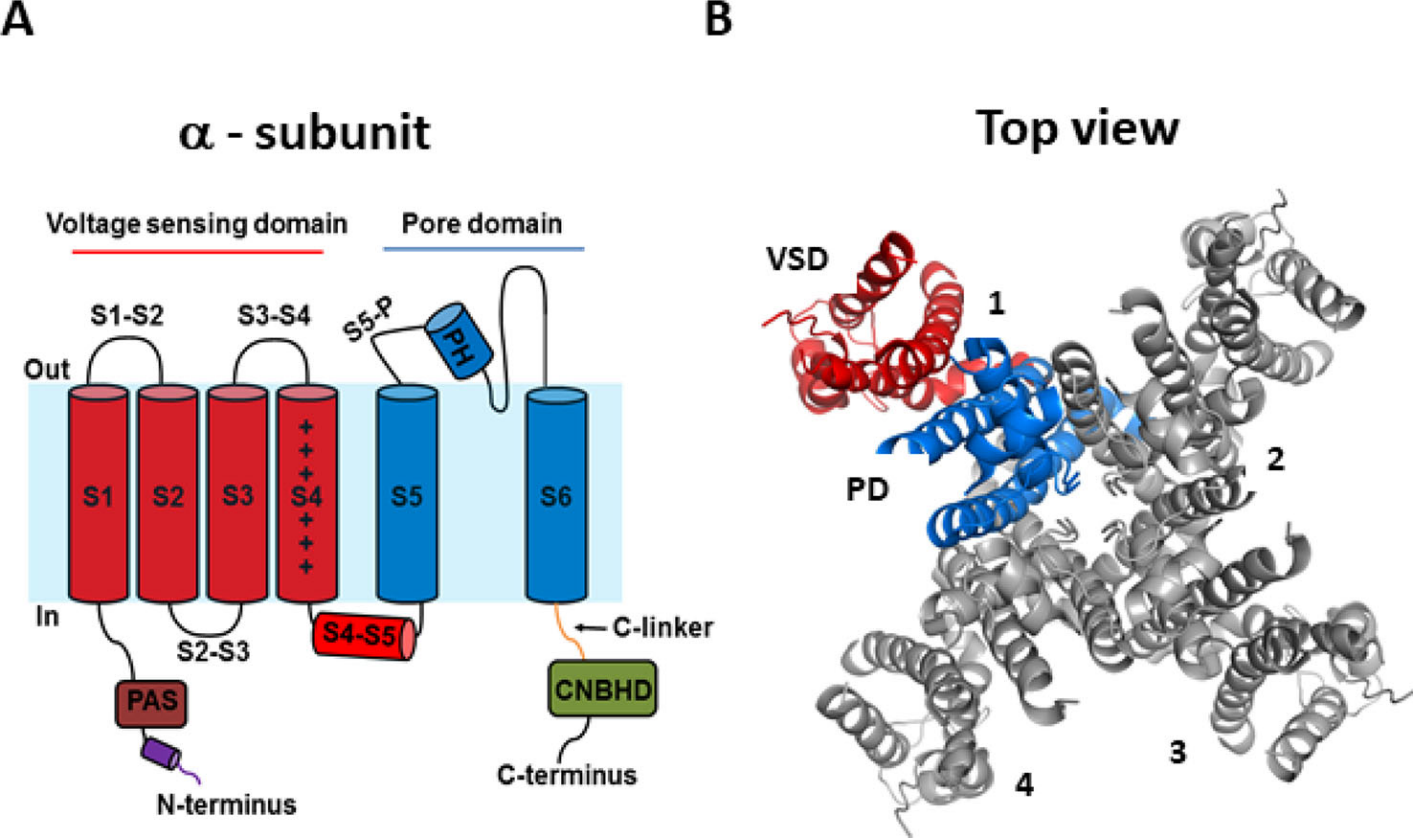
hERG channel structure. **(A)** Schematic of a single α-subunit of the hERG channel comprising of six α-helical transmembrane segments (S1–S6). The voltage sensing domain (VSD) (S1–S4) and pore domain (PD) (S5–S6) are highlighted in red and blue, respectively. Important features of the hERG channels include a short S4–S5 linker, an intracellular N-terminus containing a PAS domain shown in brown, a cap region shown in purple, and the C-terminus containing a C-linker shown in green and a cNBHD domain shown in gray. **(B)** Top view of the cryogenic electron microscopy (cryo-EM) structure of the hERG channel assembled as a tetramer with the central conducting pore formed by pore domains from all four subunits. For clarity, only one subunit is highlighted (5VA1.pdb) (Wang and MacKinnon, 2017).

Recent cryogenic electron microscopy (cryo-EM) structures of the hERG channel and the related eag1 channel in a depolarized conformation have provided direct and highly valuable insight into understanding the gating properties of hERG channels and their role in the heart and drug discovery (Whicher and MacKinnon, 2016; Wang and MacKinnon, 2017). The *KCNH2* gene encodes the pore-forming α-subunit of the voltage-gated K^+^ channel, Kv11.1, commonly referred to as hERG. The hERG channel is tetrameric, with each subunit comprised of six α-helical transmembrane segments (S1–S6) and large intracellular N- and C-terminus domains (**Figure 1**). Voltage sensitivity of the hERG channel is predominantly imparted by the S1–S4 transmembrane segments, with S4 containing six positively charged amino acids and S1–S3 segments possessing several negatively charged amino acids thought to act as counter-charges. The impetus for conformational changes leading to pore opening is the movement of the positively charged amino acids of S4 in response to the force exerted upon them by the electric field across the membrane. Since the transmembrane segments of hERG resemble those of the eag1 family member, Kv10.1, it is reasonable to presume that the three-dimensional structure of the hERG S4 segment is likely to be a 3_10_ helix (Whicher and MacKinnon, 2016), although the dynamic structure of the S1–S4 voltage sensing domain (VSD) during open to closed transitions is not known. The VSD of hERG has previously been suggested to be coupled to the pore domain (PD), formed by S5 and S6, *via* the S4–S5 linker, a short flexible connecting sequence known to be important in electromechanical coupling of VSD motions with the pore in other Kv channels (Tristani-Firouzi et al., 2002; Ferrer et al., 2006; Van Slyke et al., 2010; Cheng and Claydon, 2012; Ng et al., 2012; Hull et al., 2014). However, the recent cryo-EM structures of eag1 and hERG channels have questioned this. The structures revealed that the S4–S5 linker is a short loop that is not domain swapped (i.e., the S4–S5 linker of one subunit interacts with the C-terminal portion of the S6 helix in the same subunit, rather than the adjacent subunit) and thus, may not function as a mechanical lever. The S6 segment of hERG channels also lacks a proline-valine-proline (PVP) motif that would narrow the pore region and which, in other channels, is suggested to orient the S6 to allow it to interact with the S4–S5 linker. The lack of this structure combined with the short non-domain swapped S4–S5 linker contributes to the idea that eag family channels may have unconventional means of electromechanical coupling (Thouta et al., 2014; Lörinczi et al., 2015; Whicher and MacKinnon, 2016; Malak et al., 2017; Malak et al., 2019). An alternative model of gating translation from the VSD to the PD (Whicher and MacKinnon, 2016; Wang and MacKinnon, 2017) proposes that the deactivated (down state) of the S4 voltage sensor interacts directly with the C-linker to induce a bend in S6 to close the pore gate. The up or depolarized conformation of S4 would enable rotation of the C-linker such that it loosens the S6 thereby relieving the high-energy bend to open the pore. The involvement of the cytosolic domains in the translation of voltage sensing to the pore region is an intriguing possibility that requires further functional testing to establish whether this is the means by which VSD movement is translated to the PD in hERG channels. Certainly, the cytosolic N- and C-terminal domains play a critical role in gating of hERG channels and contain structurally well conserved domains among closely related channels (Adaixo et al., 2013). The well-structured distal N-terminus contains a Per-Arnt-Sim (PAS) domain (residues 1–136) participates in functionally important interactions with the S4–S5 linker and the C-terminus to regulate channel deactivation (Fernández-Trillo et al., 2011; Adaixo et al., 2013; de la Peña et al., 2015; de la Peña et al., 2018). The main physical feature of the hERG C-terminus is the cyclic nucleotide-binding homology domain (cNBHD) which shares homology with that in cyclic nucleotide-gated (cNG) and hyperpolarization-activated cyclic nucleotide-gated (HCN) channels. However, the cNBHD does not appear to be directly regulated by binding of cyclic nucleotides (Brelidze et al., 2012; Marques-Carvalho et al., 2012; Brelidze et al., 2013) as a result of the binding pocket being occupied by an “intrinsic ligand” (Brelidze et al., 2012; Marques-Carvalho et al., 2012; Brelidze et al., 2013; Zhao et al., 2017), which mediates interactions between the PAS domain and cNBHD to regulate channel closing (Zhao et al., 2017; Codding and Trudeau, 2019). Further structures of closed channel state would undoubtedly contribute to our understanding of the dynamic structure of hERG channels.

### Gating of the hERG Channel

**Figure 2:**
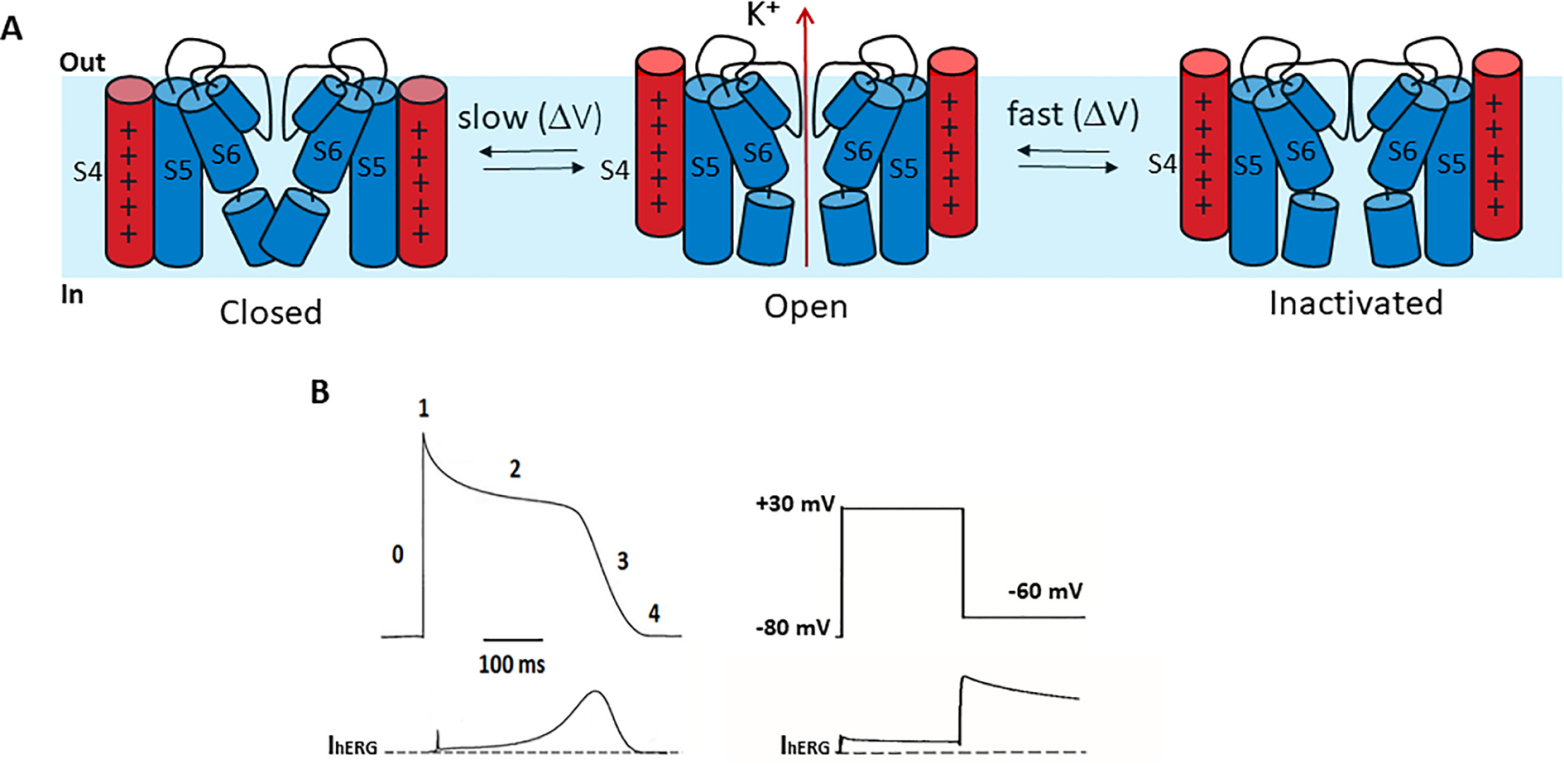
hERG channel gating. **(A)** Schematic of the hERG channel gating scheme representing the state transitions between the closed and open stated being slower than transitions between open and inactivated states. **(B)** Left. A stylized ventricular cardiac action potential waveform (top) with five distinct phases and the corresponding current conducted by hERG channels (bottom). Right. In a step voltage clamp experiment, depolarization to +30 mV activates and quickly inactivates hERG channels, producing a small outward current. Upon repolarization to −60 mV hERG channels quickly recover from inactivation, producing a large resurgent current that decays slowly due to slow deactivation.

Compared to other Kv channels, such as the archetypal *Shaker* channel, hERG channels exhibit unusual gating properties. hERG channels activate and deactivate slowly, but inactivate and recover from inactivation rapidly and with a strong dependence on voltage (Sanguinetti et al., 1995; Trudeau et al., 1995) (**Figure 2A**). The result is that upon membrane depolarization, only a small outward current is generated, because the transition from closed to open states is slow (e.g., hERG activates in ~60 ms at +60 mV, whereas *Shaker* activates in <2 ms) (Hoshi et al., 1994; Wang et al., 1997) and channels inactivate rapidly (hERG inactivates in 1–2 ms at +60 mV) (Schönherr and Heinemann, 1996; Smith et al., 1996; Spector, 1996; Rasmusson et al., 1998). Upon membrane repolarization, however, channels recover rapidly from inactivation into the open state giving rise to a resurgent current before slow channel deactivation (**Figure 2B**). This resurgent current provides a significant contribution to cardiac repolarization associated with the termination of the action potential. Despite their critical role in determining cardiac excitability, the molecular bases of these unusual gating properties remain poorly understood. Below, we briefly highlight current knowledge pertaining to each gating step, since these are reviewed in detail elsewhere (Cheng and Claydon, 2012; Vandenberg et al., 2012).

#### Activation

The physiological significance of slow hERG channel activation is a reduced channel availability and reduced repolarizing current during the early phases of the cardiac action potential. Several studies measuring gating currents and/or fluorescence reports of VSD movement suggest that the unusually slow activation in hERG channels results from slow voltage sensor movement that is the rate limiting step (Smith and Yellen, 2002; Piper et al., 2003; Piper et al., 2005; Van Slyke et al., 2010; Thouta et al., 2014), although some fraction of S4 charge appears to move rapidly (Wang et al., 2013; Goodchild and Fedida, 2014). The molecular determinants of slow VSD activation in hERG channels remains poorly understood. Like other K_v_ channels, the hERG channel contains a series of basic residues in the S4 voltage sensor that traverse the electric field upon changes in membrane voltage (Bezanilla et al., 1991; Starace and Bezanilla, 2004; Ahern and Horn, 2005) and this induces opening of the channel pore (Lu et al., 2002; Long, 2005). The hERG channel VSD comprises fewer charges than *Shaker-*like channels, with six basic residues: K525, R528, R531, R534, R537, and K538. Mutation scanning shows that mutation of K525, R528, and R531 perturbed activation gating, suggesting that these sidechains influence gating charge translocation (Subbiah et al., 2004; Piper et al., 2005). This is supported by the state-dependent accessibility of these sites to membrane-impermeable thiol-modifying reagents during activation (Zhang et al., 2004; Elliott et al., 2009). Limiting slope analysis estimates of the number of elemental gating charges moved per channel tetramer during activation (~8 e_o_) (Zhang et al., 2004) are also consistent with these observations. Interestingly, this suggests more limited movement of the hERG S4 segment than in *Shaker* channels, where MTSET accessibility studies show the translocation of four outermost arginines, consistent with the movement of ~12–14 e_o_ across the electric field (Aggarwal and MacKinnon, 1996; Seoh et al., 1996). Of the outer three hERG S4 charges, K525 is particularly important in stabilizing closed channel states in a manner that involves non-electrostatic functional interactions with a gating charge transfer center formed by F463 and D466 within the voltage sensing unit (Liu et al., 2003; Subbiah et al., 2004; Cheng et al., 2013). The presence of lysine at this site is peculiar to hERG channels and may contribute to slow gating. The presence of K538, at the base of the S4 segment alongside R537, is equally unusual and is important in stabilizing closed channel states *via* functional interactions with the gating charge transfer center (Cheng and Claydon, 2012; Cheng et al., 2013). These observations are supported by the cryo-EM hERG structure (Wang and MacKinnon, 2017) and lead to the suggestion that overcoming the energetics of stabilizing interactions between the K525 outermost S4 charge and the gating charge transfer center, and the shuttling of S4 charges through the transfer center, may limit hERG channel activation kinetics, although this remains to be directly demonstrated.

#### Inactivation

Slow activation of hERG channels is accompanied by an unusual fast inactivation process that occurs more rapidly than activation and is voltage-dependent. hERG channel inactivation is P/C-type in nature in that it is slowed in the presence of external tetraethylammonium and largely unaffected by deletion of N-terminus (Schönherr and Heinemann, 1996), and sensitive to ion occupancy of the selectivity filter (Schönherr and Heinemann, 1996) suggesting that inactivation of hERG channels involves a collapse of the selectivity filter (Smith et al., 1996). The recent hERG cryo-EM structure highlighted subtle rearrangements of the selectivity filter that might correlate with rapid hERG inactivation gating (Wang and MacKinnon, 2017). In particular, the unique position of F627 within the selectivity filter may play a key dynamic role, which may be demonstrated by future functional investigation.

The voltage-dependence of inactivation does not appear to derive from positively charged S4 residues (Zhang et al., 2004) and evidence of distinct origins of the voltage-dependence of activation and inactivation comes from an alanine scan of hERG S4 residues, which highlighted a discrete cluster of residues that perturbed inactivation when mutated without significant effect on activation gating (Piper et al., 2005). The most comprehensive description of the molecular determinants of inactivation involves a global model with complex rearrangements throughout the channel that are initiated by K^+^ efflux from the pore. In this Japanese puzzle box model a precise sequence of moves that involves interconnected but separate components is required to open and close the inactivation gate (Wang et al., 2011).

#### Deactivation

Another hallmark feature of hERG channel gating is slow deactivation, which is important for producing the resurgent current during repolarization of the cardiac action potential (Sanguinetti et al., 1995; Smith et al., 1996). Regulation of slow deactivation in hERG channels is complex, and is modulated by multiple regions of the channel. Mutations within the distal N-terminus Cap domain, consisting of residues 1–26, accelerate deactivation suggesting that this domain is essential for normal hERG deactivation gating (Cabral et al., 1998; Wang et al., 1998; Gustina and Trudeau, 2009; Muskett et al., 2011; Ng et al., 2011). The recent hERG cryo-EM structure shows that the N-terminus is indeed structurally integrated within the VSD-PD interface (Wang and MacKinnon, 2017). Consistent with this, application of a PAS domain fragment (1–135) restores slow deactivation to N-terminally deleted (Δ2–354) fast deactivating hERG channels (Cabral et al., 1998; Gustina and Trudeau, 2009; Gustina and Trudeau, 2011; Gustina and Trudeau, 2012; Gianulis et al., 2013; Gustina and Trudeau, 2013) and this is dependent on interactions between the PAS and cNBHD (Gustina and Trudeau, 2011; Gianulis et al., 2013). The N-terminus may also interact with the S4–S5 linker to regulate deactivation gating (Wang et al., 1998; Li et al., 2010; Van Slyke et al., 2010; de la Peña et al., 2011).

Slow deactivation is also influenced by the transmembrane core of the channel. Mutation of the negative S4 counter charge residues in S1–S3 all accelerated deactivation kinetics leading to the proposal for a “master-switch” in hERG channels that maintains slow deactivation (Liu et al., 2003). In this model, each of the negative residues need to be present to keep the master-switch in the ON position, and mutations or conditions that turn OFF the master-switch set the channel in a fast deactivation mode (Liu et al., 2003). A potential candidate for this master-switch has been suggested to be the PAS domain in the N-terminus (Liu et al., 2003). Another potential regulator for slow deactivation is voltage sensor relaxation and is an intrinsic property of the VSD in response to depolarization that stabilizes the activated VSD in a relaxed, stable conformation. In doing so, more energy is required to return the voltage sensor than to activate it, thus inducing a hyperpolarizing-shift in the voltage-dependence of deactivation compared to that for activation, and a slowing of the deactivation kinetics. Recent studies suggest a role for the N-terminus in stabilizing the activated voltage sensor in a relaxed state (Tan et al., 2012; Goodchild et al., 2015; Thouta et al., 2017). As an important contributor to slow deactivation, voltage sensor relaxation could perhaps act as a master-switch that is influenced by mutations within the transmembrane core, as well as the N-terminus. In the following sections, we discuss current understanding of relaxation in hERG channels and the mechanisms by which stabilizing voltage sensor transitions might be coupled to the pore to control closing.

### Hysteresis of Gating in Voltage-Dependent Ion Channels

Hysteresis is a phenomenon by which a system displays differing responses depending on its history. In other words, hysteretic behavior is thought to give a system “memory.” Such hysteretic behavior is observed in numerous chemical, physical, biological, and engineering systems. One example of the physiological importance of hysteresis is in the regulation of activation, deactivation and inactivation gating of voltage-gated Na^+^ (Nav) channels and Kv channels to control action potential firing, which has been expertly reviewed elsewhere (Villalba-Galea, 2017). Hysteresis in ion channels has also been suggested to play a critical role in the normal heartbeat (Männikkö et al., 2005), rhythmic firing of pacemaking neurons (Bruening-Wright and Larsson, 2007), the regulation of cellular excitability (Chatelain et al., 2012; Corbin-Leftwich et al., 2016), and temperature sensitization (Liu et al., 2011; Liu and Qin, 2015).

**Figure 3:**
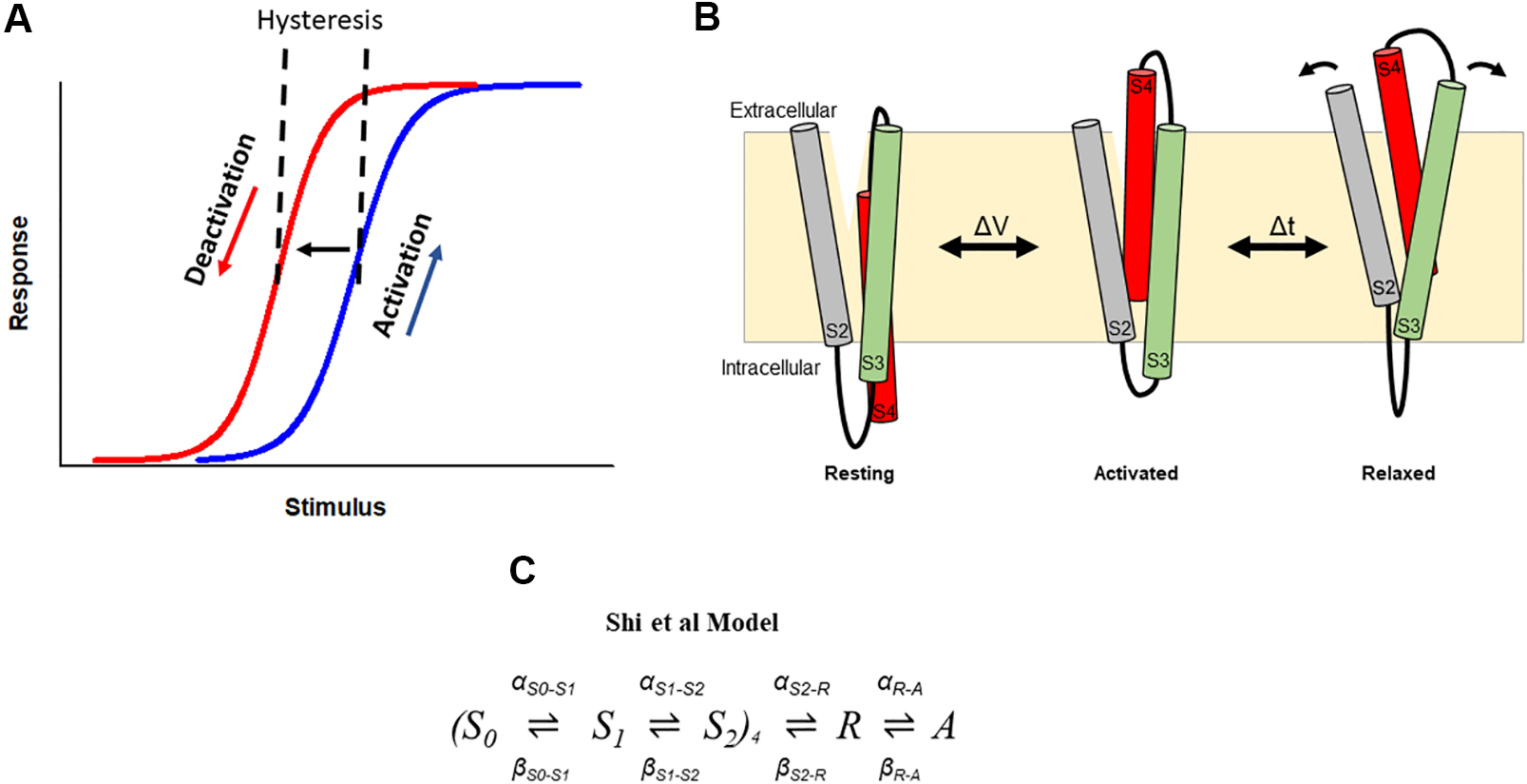
Hysteresis in hERG channels due to voltage sensor relaxation. **(A)** In a hysteretic system, the response of the system differs in response to activating and deactivating stimuli. In hERG channels, depolarization activates (blue) channels, which then shift to a different mode (i.e., enter the relaxed state), from which deactivation follows a different response upon repolarization (red). This produces a hysteresis. **(B)**. Cartoon representation of stabilization the S4 voltage sensor into a relaxed conformation during voltage sensor relaxation. Arrows represent hypothetic reorganization of helices during relaxation as a result of interactions involving acidic residues in S2–S3. **(C)**. The Shi et al. model of voltage sensor gating adopts the formulation of the Piper *et al*. gating charge model and includes addition of description of hysteresis behavior of the hERG channel voltage sensor as a result of voltage sensor relaxation (Shi et al., 2019).

Hysteresis in voltage-gated ion channels can be observed during each activation and deactivation cycle. An early observation in squid giant axon Nav channels showed a hyperpolarizing-shift of the voltage-dependence of gating charge return compared with that of gating charge activation when the membrane was held for some time at a depolarized potential (Bezanilla et al., 1982). In this case, this hysteresis behavior indicated a change in the energy landscape during deactivation such that the return of charge to rest occurred *via* an alternative kinetic pathway than that during activation. The measured observation is that the voltage sensor required stronger repolarization to return to its resting position and to close the pore gate than was required to activate it (**Figure 3A**). Similar hysteresis behavior has been reported in a broad collection of channels including sodium channels of the squid giant axon and NaChBac (Bezanilla et al., 1982; Kuzmenkin et al., 2004), calcium channels (Brum and Rios, 1987; Brum et al., 1988; Shirokov et al., 1992), potassium channels, such as KcSA (Tilegenova et al., 2017), *Shaker* (Haddad and Blunck, 2011; Lacroix et al., 2011; Labro et al., 2012; Priest et al., 2013), Kv1.2 (Labro et al., 2012), Kv3.1 (Labro et al., 2015), Kv7.2/7.3 (Corbin-Leftwich et al., 2016), Kv11.1(hERG) (Piper et al., 2003; Tan et al., 2012; Hull et al., 2014; Goodchild et al., 2015; Thouta et al., 2017; Shi et al., 2019), Kv12.1 (Dierich et al., 2018), and HCN channels (Elinder et al., 2006; Bruening-Wright and Larsson, 2007). In HCN channels, the term mode-shift has been used to describe the hysteresis in the voltage-dependence of activation and deactivation in response to prolonged depolarization (Elinder et al., 2006), and the terms hysteresis and mode-shift are often used to describe the separation between the voltage-dependence of Kv channel activation and deactivation. Here we use the term hysteresis to describe the separation of the voltage-dependence of activation and deactivation, and mode-shift to describe the mechanism for the hysteretic behavior.

The mechanistic basis that underlies hysteretic behavior in K^+^ channels has only recently been explored. Initially, the time course of development of the mode-shift was correlated with that of P/C-type inactivation, suggesting that P/C-type inactivation is required for mode-shift to occur (Bezanilla et al., 1982; Olcese et al., 1997). In KcsA channels, mode-shift of gating has also been suggested to be an intrinsic property of the PD caused by structural changes at the selectivity filter associated with P/C-type inactivation (Tilegenova et al., 2017). Other studies showed that mutations in the S4–S5 linker and S6 of *Shaker* channels which uncouple the VSD from the PD impede mode-shift, suggesting that mode-shift arises from the mechanical load placed on the voltage VSD by the PD (Batulan et al., 2010; Haddad and Blunck, 2011). However, there is also plausible evidence that mode-shift behavior is an intrinsic property of the voltage sensor. For example, hysteresis is observed in proteins that comprise a voltage sensor-like domain without a canonical pore, such as Hv1 proton channels (Villalba-Galea, 2014), *Shaker* channels with the PD deleted (Zhao and Blunck, 2016), and *Ciona intestinalis* voltage sensor-containing phosphatase (*Ci*-VSP) (Villalba-Galea et al., 2008). In the latter, a site-specific fluorophore tag reporting voltage sensor dynamics revealed a slow component of fluorescence change that is kinetically distinct from that reporting charge movement. This suggested that the voltage sensors adopt an alternate conformation following activation, which the authors termed the relaxed state of the voltage sensor (**Figure 3B**). Additional studies in *Shaker* channels demonstrated that the mode-shift is caused by prolonged depolarization, which shifts the voltage sensor into this more stable relaxed state, a process termed voltage sensor relaxation (Lacroix et al., 2011; Labro et al., 2012; Priest et al., 2013). In support of this idea, voltage sensor relaxation in *Shaker* channels is also sensitive to both the length and composition of the S3–S4 linker (Priest et al., 2013). These observations emphasize an intrinsic predilection of the voltage sensing unit to relax into a stable activated state from which S4 return requires the input of additional energy.

### Mode-Shift and Voltage Sensor Relaxation in hERG Channels

hERG channel gating displays a prominent hysteresis in the voltage-dependencies of activation and deactivation of both the movement of the voltage sensor (voltage sensor mode-shift) and the gating of the pore (ionic mode-shift). Interestingly, mode-shift in hERG channels occurs on a physiological time scale suggesting that the dynamic switching of voltage-dependencies of activation and deactivation gating may contribute to the amplitude and timing of the repolarizing I_Kr_ current during the cardiac action potential (Piper et al., 2003; Tan et al., 2012; Goodchild et al., 2015; Thouta et al., 2017; Shi et al., 2019). In *Shaker* and Kv1.2 channels, the time-dependence of voltage sensor stabilization and mode-shift were measured by applying depolarizing steps of increasing duration and observing the progressive slowing of charge return (Lacroix et al., 2011; Labro et al., 2012). Charge return slowed in a biphasic manner with the faster phase kinetically associated with pore opening and therefore was suggested to reflect stabilization of the activated voltage sensor by the open pore. The second component of charge return slowing occurred over 2–4 s and was attributed to reconfiguration of the voltage sensor into the relaxed state. In Kv3.1 channels a very rapid component of charge return that kinetically precedes pore gate opening was observed, and this was attributed to an ultra-fast relaxation mechanism in these channels (Labro et al., 2015). In hERG channels, increasing depolarization duration slows charge return (Goodchild and Fedida, 2014; Goodchild et al., 2015; Thouta et al., 2017; Shi et al., 2019). The slowing of voltage sensor return showed a biphasic dependence upon depolarizing step duration similar to that in *Shaker* and Kv1.2 channels, with a fast phase (tau = 34 ms) and a slower component (tau = 2.5 s) (Thouta et al., 2017). These data highlight two important points. The first is that the fast phase correlates kinetically with pore gate opening, which is slower in hERG than in *Shaker* channels. These findings therefore support the assertion that stabilization of the activated voltage sensor occurs in response to opening of the pore gate. The second is that the time course of the slower component of voltage sensor stabilization is similar in hERG, *Shaker*, and Kv1.2 channels despite marked differences in inactivation properties: *Shake*r and Kv1.2 inactivation occurs on the timescale of seconds and is voltage-independent, whereas hERG inactivation is strongly voltage-dependent and occurs with a tau of a few milliseconds. These observations have led to the suggestion that relaxation is unrelated to inactivation in hERG channels, an idea that is consistent with studies showing a lack of correlation between inactivation and mode-shift in hERG mutant channels that either reduce (S631A) or abolish (S620T) inactivation (Piper et al., 2003; Tan et al., 2012).

Stabilization of the activated hERG voltage sensor by the open pore and by its transition into the relaxed state most likely contributes to the observed voltage sensor mode-shift by retarding charge return upon repolarization. A similar hysteresis is observed in the voltage-dependencies of ionic current flow through the pore during activation and deactivation. One question worth considering is to what extent relaxation and stabilization of the voltage sensor contribute to these hysteretic behaviors. When voltage steps of physiological duration were applied to measure activation and deactivation, hERG channels showed a profound ionic hysteresis with the voltage-dependence of ionic current activation and deactivation separated by ~65 mV on the voltage axis. However, longer voltage step durations that allow the slow transitions to reach steady-state, produced an ionic hysteresis of ~15 mV (Thouta et al., 2017). Similar experiments capturing pseudo steady-state gating charge movement revealed a voltage sensor hysteresis of ~40 mV, much greater than that of ionic current through the pore. This observation has led to the suggestion that, upon repolarization, pore closing is energetically more favorable than the return of the voltage sensor to its resting state. The consequence of this is that the pore may close while some of the voltage sensors in the tetramer remain in their extruded position. Approximations suggest that ~25% of charge (functionally equivalent to one voltage sensor per channel tetramer) is needed to return to close the pore (Thouta et al., 2017).

Together, the evidence from hERG channels support a model in which depolarization stabilizes voltage sensors in an activated configuration producing hysteretic behavior, and that return of a single voltage sensor to the resting state is sufficient to close the channel pore gate. In this model, pore gate opening slows the return of the voltage sensor involving some form of coupling between the PD and VSD, followed by a further slower stabilization of the activated voltage sensor into the relaxed state, which is an intrinsic property of the sensor itself. In the next section, we consider evidence indicating potential structural interactions that underlie these mechanistic behaviors.

### Structural Determinants of hERG Voltage Sensor Stabilization and Relaxation

#### The S1–S2 and S3–S4 Extracellular Linkers

In *Shaker* channels the S3–S4 linker plays a role in stabilizing the activated configuration of the voltage sensor in response to prolonged depolarization (Priest et al., 2013). Reducing the length of the 31 residue linker increased the extent of voltage sensor relaxation and a role for a negatively charged cluster of residues at the N-terminal margin of the linker was also noted. Although the extent of voltage sensor relaxation has not been quantified in many channels, available data suggested a strong inverse correlation between S3 and S4 linker length and the extent of voltage senor relaxation (Priest et al., 2013). However, modifications to the hERG S3–S4 linker have little influence on mode-shift behavior (Thouta et al., 2017). Neither increasing the length, nor alteration of the amino acid composition of the short native linker, altered the hysteresis in hERG channels. Similar findings were observed with alterations to the S1–S2 linker. These observations suggest that in hERG channels the extracellular linkers do not play a role in stabilizing the activated configuration of the voltage sensor as they appear to do in *Shaker* channels. This may be due to the unique configuration of S4 gating charges as discussed below.

#### The S4–S5 Linker

In *Shaker* channels specific interactions between residues in the S4–S5 linker and the pore contribute to mode-shift behavior (Haddad and Blunck, 2011). In hERG channels, information about the influence of the S4–S5 linker on mode-shift and relaxation is less comprehensive. Such information is particularly relevant given the shorter linker length and its non-domain swapped connection of the VSD with the PD in hERG channels. Functional studies of hERG channels split within the S4–S5 linker such that tetramers are formed by independent and uncoupled VSD and PD show perturbations in deactivation gating, with less impact on activation gating (Lörinczi et al., 2015). This observation suggests that slow deactivation gating in hERG channels is incumbent upon communication involving the S4–S5 linker. This is consistent with observations that perturbations within the S4–S5 linker dramatically influence the open-closed equilibrium in hERG channels (Sanguinetti and Xu, 1999; Tristani-Firouzi et al., 2002; Ferrer et al., 2006; Alonso-Ron et al., 2008; Van Slyke et al., 2010; Hull et al., 2014). However, while mutations within the hERG S4–S5 linker are well known to accelerate deactivation gating, available evidence from G546L mutant channels shows that voltage sensor mode-shift remains intact in these channels (Hull et al., 2014). Interestingly, while increasing depolarizing durations slows voltage sensor return in G546L mutant channels, the pattern of slowing is monophasic, rather than biphasic, lacking the fast phase of slowing attributed to pore induced stabilization of the activated voltage sensor (Thouta et al., 2017). This finding leads to the suggestion that S4–S5 linker perturbation interferes with pore-to-voltage sensor coupling in the control of deactivation. Such an influence of the S4–S5 linker on the coupling between the pore gate and the activated voltage sensor is consistent with the voltage sensing mechanism suggested for eag channel gating in which the S4–S5 linker alters the interaction between S4 and the inner S6, directing S4 toward the C-linker that loosens the helical bundle and opens the pore (Whicher and MacKinnon, 2016). Thus, both functional and structural data portray a key role for the S4–S5 linker in communicating between the pore and the voltage sensor to control hERG channel deactivation gating. These findings may also be interpreted to suggest that the mechanisms of voltage sensor stabilization by the open pore gate and by relaxation are separable, and also that pore gate stabilization of the voltage sensor contributes minimally to the observed hysteresis in the voltage-dependencies of voltage sensor activation and return in hERG channels.

#### The Voltage Sensor Domain (S1–S4)

Outward translocation of the hERG voltage sensor upon activation involves the formation of stabilizing electrostatic interactions between basic residues in S4 and extracellular acidic counter charges in S1 (D411), S2 (D456 and D460), and S3 (D509) (Liu et al., 2003; Zhang et al., 2005; Piper et al., 2008). Double mutant cyclic analysis revealed energetic coupling between R531 and D456, D460, and D509 during activation and with D411 and D466 through a cooperative ionic-pairing interaction mechanism (Piper et al., 2008). Accessibility studies have also revealed that D460 and D509 stabilize the activated state (Liu et al., 2003), which is consistent with observations that neutralization of any of the acidic charges accelerates deactivation kinetics, likely by disrupting electrostatic interactions with S4 basic residues (Liu et al., 2003; Fernandez et al., 2005; Piper et al., 2008; Shi et al., 2014). Interestingly, disruption of the electrostatic pairing involving D509, either by protonation or alanine substitution, destabilizes the relaxed state of the voltage sensor leading to the loss of hysteresis and accelerated deactivation (Shi et al., 2019). This suggests that external acidic residues form stabilizing electrostatic interactions that are critical in recruiting the voltage sensor into the relaxed conformation and that these consequently control pore closure. Taken together with the observed role of the S4–S5 linker in coupling pore motions to stabilization of the S4 described in the previous section, these findings indicate dual regulation, i.e., at both extracellular and intracellular ends, of S4 movement that contributes to mode-shift behavior in hERG channels and their unique slow deactivation properties. Further studies will determine whether all S4 counter charges play a similar stabilizing role, as well as the specific roles played by each S4 gating charge.

#### The N-Terminus

The N-terminus is well recognized as an integral component of deactivation gating in hERG channels that stabilizes the open state of the channel (Cabral et al., 1998; Wang et al., 1998; Wang et al., 2000; Muskett et al., 2011; Ng et al., 2011; Gustina and Trudeau, 2011). However, the mechanism by which this occurs still lacks definition. Using voltage clamp fluorimetry (VCF) to track voltage sensor movement, Tan et al. showed that while deletion of the distal N-terminus (or even single point mutations, R4A, R5A, G6A) accelerated voltage sensor return upon repolarization, perturbation of the N-terminus did not alter mode-shift of the voltage sensor (Tan et al., 2012). This finding is consistent with an intrinsic mechanism of voltage sensor stabilization and that coupling of the pore with the voltage senor was disturbed by the N-terminal perturbations. Indeed, the authors proposed that the N-terminus serves as an adaptor, mediating communication between the voltage sensor and pore gate. Initial measurements of voltage sensor movement from gating currents reported that N-terminus perturbations might reduce voltage sensor mode-shift (Goodchild et al., 2015); however, subsequent longer duration recordings enabling steady-state measurements showed that the mode-shift of gating charge movement in hERG channels lacking the distal N-terminus (Δ2–135) is similar to that in WT channels (Thouta et al., 2017). In this study, slowing of charge return upon increasing depolarizing step duration measured in Δ2–135 channels showed a biphasic function with similar early and late components of charge return slowing to that seen in full-length channels. These observations suggest that both the stabilization of the voltage sensor by the open pore gate and the intrinsic voltage sensor relaxation process are preserved in hERG channels lacking the N-terminus. It is a clear and consistent observation, however, that perturbation of the N-terminus enhances the kinetics of voltage sensor return (Tan et al., 2012; Goodchild et al., 2015; Thouta et al., 2017). This observation supports the idea proposed by Tan et al. that closure of the pore during deactivation can be modified at the interface of the voltage sensor and pore gate by the N-terminus independent of changes in voltage sensor configuration and demonstrates that coupling of the voltage sensor with the pore gate is an important mediator of deactivation in addition to relaxation of the voltage sensor (see section below on hERG activator compounds) (Tan et al., 2012).

### Targeted Modulation of hERG Voltage Sensor Relaxation

#### The Relaxed State of the Voltage Sensor Is Destabilized by Extracellular Protons

hERG channel deactivation kinetics are sensitive to changes in the extracellular proton concentration and numerous studies have shown that acidosis accelerates deactivation rate (Anumonwo et al., 1999; Bérubé et al., 1999; Jiang et al., 1999; Terai et al., 2000; Bett and Rasmusson, 2003; Du et al., 2010; Zhou and Bett, 2010; Du et al., 2011; Van Slyke et al., 2012; Shi et al., 2014; Shi et al., 2019). pH reductions within the physiological range (as low as pH 6.5) accelerated both the fast and the slow components of deactivation with a *pKa* of ~6.8 (Bett and Rasmusson, 2003; Shi et al., 2019) with little effect channel conductance, and other voltage-dependent gating parameters (Jiang et al., 1999; Bett and Rasmusson, 2003; Shi et al., 2019). Until recently, the mechanism by which protons modulate deactivation was unknown although studies had ruled out involvement of extracellular histidine residues (Jiang et al., 1999; Van Slyke et al., 2012), the N-terminus, and P/C-type inactivation, and shown the action to be mediated by an extracellular site (Jiang et al., 1999).

Measurements of voltage sensor relaxation from fluorescence reports of voltage sensor movement and gating current recordings recently provided a plausible mechanism for the action of protons on hERG channel deactivation (Shi et al., 2019). These studies showed that acidic pH (pH 6.5) abolished voltage sensor mode-shift behavior. In particular, elevated external protons produced a depolarizing-shift of the voltage-dependence of the return of the voltage sensor upon repolarization without altering that of voltage sensor activation. This evidence suggested that the relaxed conformation of the hERG voltage sensor was destabilized by protons, such that less energy was required to return the voltage sensor to rest leading to an acceleration of pore gate closure at a given voltage during deactivation (Shi et al., 2019).

A key site of action for this effect of external protons may be three acidic residues, D456, D460, and D509, which form a cation binding pocket that coordinates Zn^2+^, Mg^2+^, Ca^2+^, Cd^2+^, as well as H^+^ (Ho et al., 1998; Jo et al., 1999; Fernandez et al., 2005; Lin and Papazian, 2007; Abbruzzese et al., 2010; Kazmierczak et al., 2013; Shi et al., 2014; Shi et al., 2019). These sites may also interact with positive S4 gating charges as discussed in an earlier section. Consistent with a role for this site, neutralization of D509 abolished voltage sensor mode-shift at pH 7.4 mimicking the effect of external protons, which exerted no further effects on the voltage-dependence or kinetics of voltage sensor gating (Shi et al., 2019). This suggested that protons destabilize the relaxed state of the voltage sensor by disrupting electrostatic interactions formed between D509 and basic residues in the S4 voltage sensor. In the hERG cryo-EM structure, D509 is located at the external end of S3 close to the unresolved flexible S3–S4 linker, and readily accessible to extracellular aqueous solvents (Liu et al., 2003). Unlike in *Shaker* and *Ci*-VSP, where entry into the relaxed state may be tracked during long depolarizations as a slow fluorescence change from fluorophores attached at the top of the voltage sensor (Villalba-Galea et al., 2008; Haddad and Blunck, 2011), voltage sensor relaxation in hERG channels is not associated with overt fluorescence reports or gating charge. Therefore, entry into the relaxed state may involve subtle reconfiguration of the hERG voltage sensor that is not associated with charge movement across the membrane electric field or significant environment change experienced by a fluorophore label attached to the top of S4. Instead, transition into the relaxed state may involve, for example, widening of a water-filled cleft at the extracellular face of S2, S3, and S4 transmembrane segments, where water molecules may act to bridge H-bond or electrostatic interactions between D509 and positive charges in the S4 to stabilize the relaxed state. Higher resolution structures near the extracellular portion of S4 will undoubtedly contribute to better understanding of this as a possible mechanism. Similarly, information on the involvement of the other two acidic residues, D456 and D460, in stabilization of the relaxed voltage sensor will also contribute to a more complete picture. Moreover, given that divalent cations share a similar site of action, it is quite plausible that such ions would, like protons, destabilize relaxation. Indeed, various divalent ions are known to accelerate deactivation kinetics (Anumonwo et al., 1999; Fernandez et al., 2005; Lin and Papazian, 2007; Abbruzzese et al., 2010; Shi et al., 2014).

#### hERG Channel Activators May Alter Coupling Between the Voltage Sensor and Pore Gate

hERG channels are highly promiscuous in their ability to bind and be blocked by a wide range of drugs with varied structure and function. This causes acquired long QT syndrome (LQTS), and presents a significant challenge to new drug development. However, this promiscuity has led to the discovery and development of potential therapeutic activator compounds that aim to enhance or rescue lost hERG channel function as a result of inherited or acquired LQTS (Kang et al., 2005; Perry et al., 2007; Perry and Sanguinetti, 2008; Wu et al., 2016). A number of such small molecule compounds have been identified and some, such as RPR260243, niflumic acid, and ginsenoside Rg3, slow hERG deactivation gating (known as Type 1 activators) resulting in increased hERG current during cardiac repolarization. RPR260243, slows deactivation kinetics by interacting with L553 and F557 at the cytosolic end of S5 and S6 (Kang et al., 2005; Perry et al., 2007; Perry and Sanguinetti, 2008). In doing so, RPR260243 likely stabilizes the open conformation of the pore leading to slower deactivation (Gardner and Sanguinetti, 2015; Wu et al., 2015). Gating current measurements show that RPR260243 had no effects on the kinetics and voltage-dependence of hERG voltage sensor movements (Abbruzzese et al., 2010), suggesting that the activator exerts its effect by altering the coupling between the voltage sensor and pore closure during membrane repolarization. Thus, these findings further support the VSD-PD interface as a critical site of modulation of deactivation gating. Interestingly, another activator, NS1643, which is a type 2 compound that increases hERG current primarily by inducing a depolarizing-shift of the voltage-dependence of inactivation, may interact within the voltage sensing unit (Guo et al., 2015). Molecular simulations placed NS1643 close to L529 in S4 in a position that could plausibly interfere with S4 gating charge stabilizing interactions with the gating charge transfer center. It remains to be seen whether NS1643 modifies the stability of the relaxed voltage sensor configuration. As such, there may be therapeutic potential in rational design of small molecule activators of hERG channels that target sites known to modulate voltage sensor relaxation and/or its coupling to the pore gate to safely restore hERG channel dysfunction.

### Kinetic Modeling of hERG Channel Relaxation

One of the earliest kinetic models constructed to describe I_Kr_ current in ventricular myocytes used first-order Hodgkin and Huxley formalism with the absence of an inactivated state (Zeng et al., 1995). Shortly thereafter, a model describing hERG channel activation and inactivation properties was developed (Wang et al., 1997), and the robustness of this Wang model is demonstrated by its continued usage. The Wang model was adopted to model I_Kr_ in the ten Tusscher human ventricular action potential waveform (ten Tusscher, 2003), although there have been several subsequent variations/refinements of the linear model, most notably to include a direct transition from the last closed state directly to the inactivated state (Clancy and Rudy, 2001; Mazhari et al., 2001; Oehmen et al., 2002). A detailed comparison of available models suggested that the most informative model was the original 5-state linear Wang model, since the forward rate of the final closed state to inactivated state transition is so low in other models that is it not required (Bett et al., 2011). Piper et al. provided the first Markov model to describe hERG channel gating current kinetics (Piper et al., 2003). This study showed that hERG on- and off-gating currents present a fast (0.5 ms) and a slow (53 ms) component. To model this behavior, two transitions, S0–S1 (fast component) and S1–S2 (slow component), were used in the gating scheme and these were treated independently for the four subunits with a positive cooperativity factor incorporated for the second transition, prior to channel opening. A derivation of this model was also used to simulate and recapitulate gating currents in the presence of Cd^2+^ (Abbruzzese et al., 2010).

Recently, a similar model of gating current kinetics was used to describe hERG channel voltage sensor relaxation (Shi et al., 2019) (**Figure 3C**). In this model, two independent transitions per subunit are followed by a voltage-dependent concerted transition to the activated state, and a subsequent voltage-independent transition into the relaxed state. The model recapitulates the main features of hERG gating currents including voltage sensor mode-shift behavior. Moreover, acceleration of the rate out of the relaxed state to mimic the destabilization of relaxation observed at low pH, selectively abolished mode-shift behavior without other gating consequences, recapitulating the experimentally observed voltage sensor behavior. From this model, acceleration of de-relaxation, or exit from relaxed state, was sufficient to reduce voltage sensor mode-shift and supported the hypothesis that destabilization of the relaxed state of the voltage sensor may drive voltage sensor return leading to accelerated deactivation (Shi et al., 2019). One limitation to this model is the absence of description of ionic activation and inactivation gating of the channel, which are needed to develop a more complete model of gating transitions of the hERG channel voltage sensor in conjunction with pore gating during voltage sensor stabilization and relaxation. This might involve an approach used previously in *Shaker* and Kv1.2 channels to construct models that describe transitions of voltage sensor domains corresponding to those of the pore (Labro et al., 2012), which may be applicable for adaptation into a hERG scheme.

## Summary

Hysteresis of hERG channel voltage-dependent gating and the role of dynamic changes in voltage-sensitivity in the control of physiological function is only beginning to be uncovered. In this review we have discussed current knowledge pertaining to the mechanistic determinants of hysteretic behavior in hERG channels and how this might influence deactivation gating and therefore resurgent repolarizing current during the cardiac action potential. We have discussed the role of different channel regions in precipitating reconfiguration of the voltage sensor into a stabilized relaxed state and highlighted the importance of an extracellular site within the voltage sensor that contributes to this stabilization. We have also discussed evidence suggesting that intracellular modulators that regulate deactivation kinetics may do so by altering coupling between the voltage sensor and pore gate during repolarization. These discussions highlight what is known about the complex interactions that regulate movement of the voltage sensor and its coupling to the pore in the control of the physiologically critical slow deactivation of hERG channels and how they might be manipulated for potential therapeutic benefit. The discussion also highlights that much remains to be discovered about how the dynamic structural stability of the voltage sensor is modulated and influenced by a complex combination of extracellular ionic interactions and intracellular coupling to the pore. It remains to be determined, for example, how these complex interactions contribute to the slow activation of hERG channels, the fast and slow components of ON gating current, and the fast and slow components of channel deactivation. Further structural information, for example from closed hERG channels, will undoubtedly provide further mechanistic insight, as would functional studies investigating voltage sensor dynamics using fluorophore reporters or gating charge translocation measurements in isolated voltage sensor domains and comparing this with dynamics in VSD-PD coupled channels. Such future research will help to understand the complex structural determinants of hERG channel gating.

## Author Contributions

YS and ST contributed equally. YS, ST, and TC wrote and approved the manuscript.

## Funding

This work was supported by a Canadian Institutes of Health Research Project Grant (PJT-156168) held by TC. Open access funds were provided by Simon Fraser University.

## Conflict of Interest

The authors declare that the research was conducted in the absence of any commercial or financial relationships that could be construed as a potential conflict of interest.
